# Three-Dimensional ERT Advanced Detection Method with Source-Position Electrode Excitation for Tunnel-Boring Machines

**DOI:** 10.3390/s24103213

**Published:** 2024-05-18

**Authors:** Shuanfeng Zhao, Bo Liu, Bowen Ren, Li Wang, Zhijian Luo, Jian Yao, Yunrui Bai

**Affiliations:** College of Mechanical Engineering, Xi’an University of Science and Technology, Xi’an 710054, China; liubo@stu.xust.edu.cn (B.L.); 22205224133@stu.xust.edu.cn (B.R.); wanglixust@stu.xust.edu.cn (L.W.); 21205016037@stu.xust.edu.cn (Z.L.); 21205016032@stu.xust.edu.cn (J.Y.); 20105016009@stu.xust.edu.cn (Y.B.)

**Keywords:** advanced detection, shield tunnel, cutterhead source electrode, 3D-ERT, physics simulation experiment

## Abstract

Tunnel-boring machines (TBMs) are widely used in urban underground tunnel construction due to their fast and efficient features. However, shield-tunnel construction faces increasingly complex geological environments and may encounter geological hazards such as faults, fracture zones, water surges, and collapses, which can cause significant property damage and casualties. Existing geophysical methods are subject to many limitations in the shield-tunnel environment, where the detection space is extremely small, and a variety of advanced detection methods are unable to meet the required detection requirements. Therefore, it is crucial to accurately detect the geological conditions in front of the tunnel face in real time during the tunnel boring process of TBM tunnels. In this paper, a 3D-ERT advanced detection method using source-position electrode excitation is proposed. First, a source-position electrode array integrated into the TBM cutterhead is designed for the shield-tunnel construction environment, which provides data security for the inverse imaging of the anomalous bodies. Secondly, a 3D finite element tunnel model containing high- and low-resistance anomalous bodies is established, and the GREIT reconstruction algorithm is utilized to reconstruct 3D images of the anomalous body in front of the tunnel face. Finally, a physical simulation experiment platform is built, and the effectiveness of the method is verified by laboratory physical modeling experiments with two different anomalous bodies. The results show that the position and shape of the anomalous body in front of the tunnel face can be well reconstructed, and the method provides a new idea for the continuous detection of shield construction tunnels with boring.

## 1. Introduction

In recent years, the number and scale of urban tunnel construction projects have increased. The shield method is widely used in urban tunnel construction projects because of its advantages of a high degree of automation, fast excavation speed, and comprehensive economic benefits. However, the increasingly complex geological environment has brought great uncertainty to the use of the urban shield method in construction, significantly increasing the difficulties and dangers of tunnel construction [1]. When carrying out shield-tunnel construction, a variety of adverse geological conditions, including karst, faults, and unlimited groundwater recharge, may be encountered. Blind excavation can lead to sudden disasters, such as unforeseen water surges, collapse, and equipment damage [2], and can even lead to catastrophic consequences such as massive property losses and casualties [3,4]. Therefore, the early detection of adverse geological conditions at the front of the tunnel face of a shield tunnel can effectively prevent the occurrence of geological disasters and ensure the safe and efficient excavation of shield tunnels [5].

The physical-exploration method is a commonly used advanced detection method in shield-tunnel construction, principally including the geo-radar method [6], the TSP tunnel seismic-wave method [7], and the transient electromagnetic method [8]. However, these geophysical prospecting methods have obvious limitations in the shield-tunnel-construction environment where undesirable geological bodies require continuous monitoring in real time. The geological radar method requires cables and antennas to be laid in front of the tunnel-boring machine during detection, which imposes certain requirements on the construction space [9]. The TSP tunnel seismic-wave method requires the use of blasting or vibration sources when exciting seismic sources, which increases the complexity and cost of construction and may also cause safety hazards [10]. The transient electromagnetic method usually experiences interference from the large number of metal structures and equipment at the shield-tunnel construction site, affecting the reliability and accuracy of the signal. In contrast, electrical resistance tomography (ERT) can effectively depict the geological structure ahead through the measurement of changes in the rock resistivity. In general, the physical properties, such as the resistivity of the surrounding rock, will be significantly different when those properties include undesirable geological bodies such as water and caves. In addition, the location, scale, shape, etc., of undesirable geological bodies also have a great impact on the conductive properties of surrounding rock [11]. Therefore, from the perspective of physical parameters, the use of electrical resistance tomography for geological advanced detection has advantages that other detection methods cannot match.

Electrical resistance tomography (ERT) imaging has attracted much attention as a non-invasive detection technique in different fields. In the field of biomedical engineering, for example, ERT technology is used for the image reconstruction of human lung pathology [12], exploration of soft tissue organs [13], and monitoring of muscle activity [14]; in the industrial field, ERT technology is used to monitor the reaction processes of various media in different pipelines and tanks [15] as well as the flow-pattern identification of a gas–liquid two-phase flow [16,17]; in the field of geophysical sciences, the ERT technique is commonly used to examine the distribution of underground geotechnical structures [18] and the distribution of geomorphic features in unknown areas [19]. Mi Kyung Park [20] used ERT technology to examine the spatial distribution and shape of underground caves in a karst area in Korea, and the Ocean University of China [21] used ERT technology to investigate the seawater–groundwater exchange process. Electrical resistance tomography technology has extensive development and application prospects in the field of geological exploration due to its advantages of visualization, simple measurement system, low cost, and fast responses. However, the majority of the existing ERT geological detection methods are two-dimensional electrical resistance tomography or pseudo-3D electrical resistance tomography. Two-dimensional ERT is limited to the geological information of a single cross-section. Pseudo-three-dimensional ERT requires multiple detections to form the three-dimensional geological information, and the geological information provided is not comprehensive. The majority of the detection methods based on ERT technology are conducted by arranging electrode arrays on the ground, placing electrodes in tunnel boreholes, or arranging survey lines behind the tunnel [22,23]. This results in the detection device requiring manual adjustment as the excavation working face advances, which is time-consuming, labor-intensive, and affects the construction progress. It is, therefore, difficult to meet the increasing scale and difficulty of the shield-tunnel project.

In view of this, this study proposes a 3D-ERT advanced detection method with source-position electrode excitation for tunnel-boring machines. This proposes a new concept for detection without stopping work. First of all, combined with the characteristics of shield construction, the tunnel-boring machine’s cutterhead-source-position electrode array was designed, which provides the data for determining the size, morphology, and location of the undesirable geological body in front of the tunnel face. Then, a numerical simulation was performed using the obtained data, and 3D inversion imaging of the undesirable geological body in front of the tunnel face was conducted. The shape and location of the undesirable geological body were determined through the inversion results. Finally, a physical simulation experimental platform was established, and the feasibility and effectiveness of the method were verified via an indoor flume-simulation experimental study, which provides a reliable experimental basis for further research and the application of advanced detection in urban shield tunnels.

## 2. Method

### 2.1. Fundamental Principle

**Figure 1:**
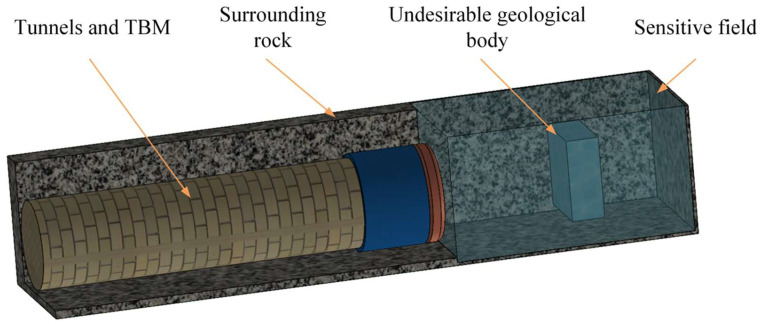
Schematic diagram of the advanced detection model.

In shield-tunnel construction, different targets in the field in front of the tunnel face have different resistivity characteristics, which represents the essential ability of ERT technology that can be used for advanced detection in shield tunnels. The basic principle of electrical resistance tomography is described as follows: first, using the conductive property differences between the surrounding rock and the surrounding undesirable geological bodies, through the measurement of the resistivity distribution in the detection area, the spatial distribution pattern of the geoelectrical field in the measured area is obtained. Second, an image reconstruction algorithm is used for three-dimensional reconstruction. A schematic diagram of the advanced detection model is shown in Figure 1. The specific implementation process is as follows: The source-position electrode array integrated on the cutterhead of the TBM is constructed, and a stable current field is established through the application of an appropriate excitation current at the boundary of the measured field through the electrode array. If the macroscopic lithological characteristics of the geological structure are changed, the resistivity characteristics of the interior will also change, and the voltage value at the measured boundary will also be changed. The electrical signal data at the boundary of the measured area are collected, and the image inversion algorithm is used to perform three-dimensional inversion imaging of the undesirable geological bodies to achieve the advanced detection of undesirable geological bodies in front of the tunnel face.

### 2.2. Forward Problem

The forward problem of the 3D-ERT advanced detection system with source-position electrode excitation is the process of solving the potential distribution in the sensitive field by knowing the space distribution of the conductivity in the sensitive field, so as to obtain the voltage data at the boundary of the sensitive field. ERT technology belongs to the category of an electrostatic field, which can be analyzed by using the electromagnetic theory, and Maxwell’s equations, as a theoretical basis of the macroscopic electromagnetic phenomena, can be used to explain the laws between the electric field and the magnetic field and between the electric charge and the electric current [24].

The differential form of Maxwell’s system of equations is as follows:

(1) $$\left\{\begin{array}{l} \nabla \cdot B=0\\ \nabla \cdot D=\rho \\ \nabla \times E=-\frac{\partial B}{\partial t}\\ \nabla \times H=J+\frac{\partial D}{\partial t} \end{array}\right.$$

Within the sensitive field of each isotropy, the constitutive equations are expressed as follows:

(2) $$\left\{\begin{array}{l} B=\mu H\\ D=\varepsilon^{*}E\\ J=\sigma E \end{array}\right.$$

where B is the magnetic induction, J is the current density, H is the magnetic field strength, D is the electric flux density, E is the electric field strength, and μ, $\varepsilon^{*}$, and σ denote the magnetic permeability, dielectric constant, and electrical conductivity, respectively.

According to Maxwell’s equation and the assumption of quasi-static field [25], the current density J at any point within the solved sensitive field satisfies the following:

(3) ∇⋅J=0

Also due to the following:

(4) E=−∇Φ

where Φ is the potential distribution in the field, which can be obtained from the above formula:

(5) $$\nabla \cdot \left(\sigma \nabla \Phi \right)=0$$

(6) $$\nabla \sigma \cdot \nabla \Phi +\sigma \nabla^{2}\Phi =0$$

The above equation is the mathematical model of ERT sensitive field. Its solution satisfies the second type of boundary conditions (Neumann conditions):

(7) $$\sigma \cdot {\left.\frac{\partial \Phi }{\partial n}\right|}_{s}=J$$

where n is the vector in the direction normal to the outside of the boundary and s is the field boundary of ERT, the problem often uses the following integral form:

(8) $$\left\{\begin{array}{l} \int_{E^{+}}\sigma \cdot \frac{\partial \Phi }{\partial n}ds=+I\\ \int_{E^{-}}\sigma \cdot \frac{\partial \Phi }{\partial n}ds=-I \end{array}\right.$$

When solving the forward model, due to the fact that the finite-element method has strong adaptability to the shape of the sensitive field and is able to deal with the inhomogeneous distribution of the medium conductivity inside the sensitive field as well as all types of linear and nonlinear problems in the electromagnetic field, this paper establishes the different types of simulation models required for the study through the finite-element method in combination with EIDORS [26] as a way of ensuring the accuracy of the study.

### 2.3. Inverse Problem

For the 3D-ERT advanced detection system with source-position electrode excitation, the inverse problem is that of solving the conductivity distribution in the measurement field with known boundary conditions of the measurement area, where the key objective is image reconstruction. Traditional image reconstruction and inversion algorithms are divided into two types: iterative algorithms and non-iterative algorithms. The iterative algorithm has a high imaging accuracy, but the imaging speed is slow; meanwhile, the non-iterative algorithm has a fast imaging speed but low imaging accuracy. However, traditional image reconstruction algorithms are usually suitable for two-dimensional image reconstruction. In this study, the image reconstruction of the undesirable geological body in front of the tunnel face is carried out using the GREIT inversion algorithm [27]. The GREIT algorithm reconstructs images with good uniformity and high quality. In actual situations, the imaging time is extremely short, which meets the needs of real-time detection of undesirable geological bodies. It uses nonlinear optimization methods to solve the inverse problem in ERT, resulting in more accurate conductivity images. The algorithm updates the reconstructed image using an iterative process based on a cost function via the transformation of the inverse problem of ERT into an optimization problem, using prior information. The objective function is as follows:

(9) x=Ry

where x is the desired conductivity distribution, y is the boundary voltage variation, and R is the reconstruction matrix. The GREIT algorithm first defines a series of training objectives $t^{\left(i\right)}$ and then seeks the R that minimizes the error of Equation (10) as the optimized reconstruction matrix, as shown in Equation (11):

(10) $$\varepsilon^{2}\left(R\right)=E_{w}\left[{\left\|x-Ry\right\|}^{2}\right]$$

(11) $$R=E_{w}\left[xy^{T}\right]{\left(E_{w}\left[yy^{T}\right]\right)}^{-1}$$

where ε is the image reconstruction error and w is a weighting matrix representing the weight of each target in the training target $t^{\left(i\right)}$. When the training targets are defined as distributions [28], the training target $t^{\left(i\right)}$ satisfies the following relationship with the conductivity distribution x and the boundary voltage variation y:

(12) x=Nt

(13) y=Mt+n

The mapping matrix between the training target $t^{\left(i\right)}$ and the ideal conductivity distribution x is N, M is the sensitivity coefficient matrix, which is obtained by solving Equations (11)–(13) jointly:

(14) $$R=N\sum_{t}^{*}M^{T}{\left(M\sum_{t}^{*}M^{T}+\lambda \sum_{n}\right)}^{-1}$$

where $\sum_{t}^{*}$ is the covariance of the training target $t^{\left(i\right)}$ and $\sum_{n}$ is the covariance of the noise. From the above equation, it can be seen that the core of GREIT lies in the mapping matrix N, which is used to map the training target $t^{\left(i\right)}$ to an ideal conductivity distribution x with good uniformity.

### 2.4. Source-Position Electrode Array Design

**Figure 2:**
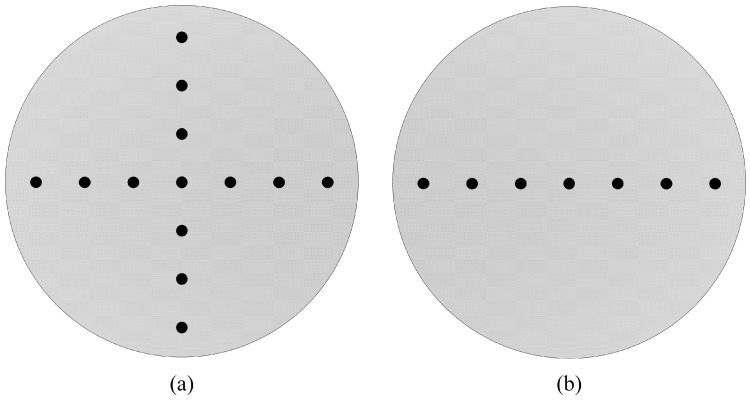
Different arrangements of the source-position electrode array. (**a**) The cross-shaped electrode array; (**b**) The one-line electrode array.

The available observation space in a shield tunnel is small, and with traditional electrode arrays, the electrodes can usually only be arranged on the side walls of the tunnel cavity and the narrow tunnel face. The tunnel-boring machine cutterhead is mainly composed of a hollow area and a main beam [29]. The electrode array can only be arranged in the main beam panel area. However, the cutters on the panel occupy further panel space. This study designed two electrode arrays based on the characteristics of the tunnel-boring machine cutterhead. The two electrode array arrangements are a cross-shaped electrode array and a one-line electrode array. The one-line electrode array has seven electrodes, and the cross-shaped electrode array has thirteen electrodes, as shown in Figure 2.

**Figure 3:**
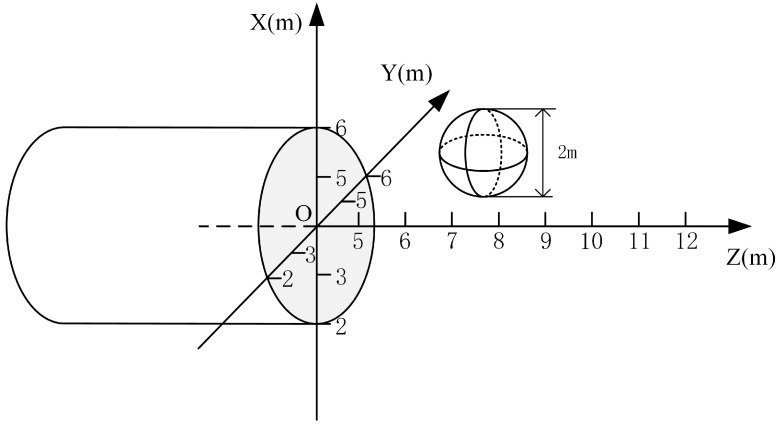
Schematic diagram of the location of the high-resistance sphere.

A three-dimensional geoelectric field finite-element model of the tunnel was established to analyze and study the different electrode array arrangements. The entire model is designed to be 12 m × 8 m × 8 m. The TBM model and the excavation tunnel are set as a whole, with a length of and a radius of 1 m. A high-resistance ball with a radius of 1 m is set on the upper-right side in front of the tunnel face. The *X*-axis denotes the height, the *Y*-axis denotes the width, and the *Z*-axis denotes the direction of the tunnel excavation, and the location schematic is shown in Figure 3.

**Figure 4:**
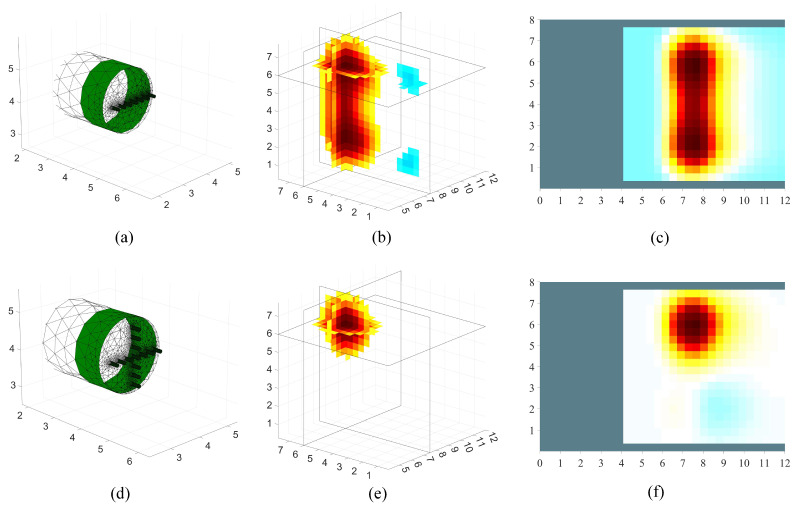
Different electrode array arrangements and reconstruction results. (**a**,**d**) are the one-line electrode array arrangement and the cross-shaped electrode array arrangement; (**b**,**e**) are the three-dimensional reconstruction results; (**c**,**f**) are the two-dimensional slices along the *Y*-axis direction past the center of the high-resistance sphere.

The three-dimensional reconstruction was performed on the established finite-element model, and the reconstruction results are shown in Figure 4. Observing Figure 4b,c, it can be clearly seen that high resistivity artifacts are formed on the other side of the reconstruction target; meanwhile, from Figure 4e,f, the high resistance ball in front of the tunnel face can be clearly seen. This shows that the arrangement of the one-line electrode array will produce a symmetrical effect. The reason for this may be that when the current is injected into the geology in front of the tunnel face through the supply electrode, the electric field formed has a certain symmetry in the vertical direction. The symmetry leads to similar propagation paths of the electric field above and below the tunnel face; therefore, an undesirable geological body located above may be incorrectly mapped below during inversion imaging. In practical engineering applications, this symmetry effect can cause engineers to misjudge the undesirable geological body in front of the tunnel face, which makes the excavation project stop abruptly and can seriously affect the construction progress. Therefore, in this study, the cross-shaped electrode array was selected as the source-position electrode array arrangement for the tunnel-boring machine’s cutterhead.

### 2.5. Measurement Strategy

**Figure 5:**
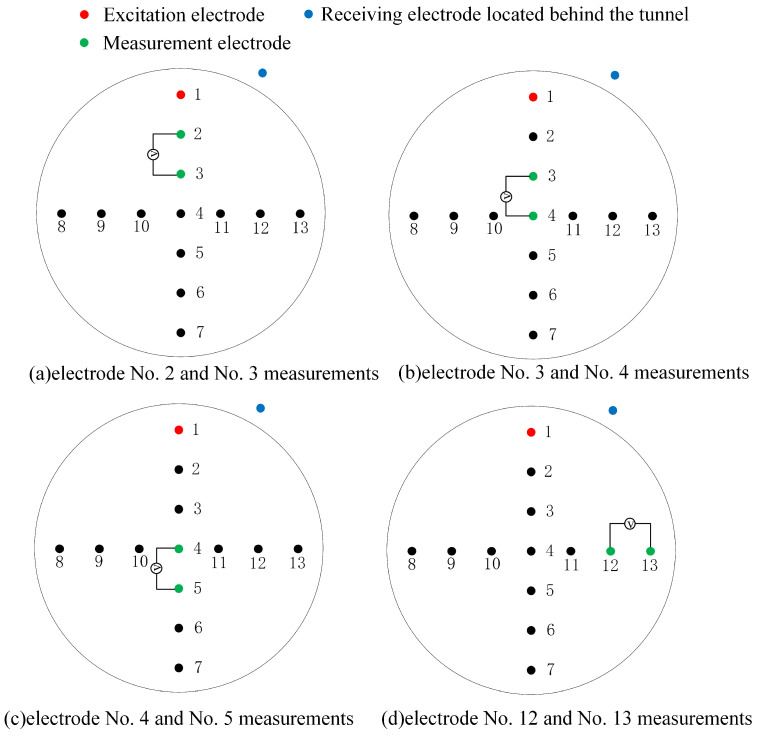
Schematic diagram of the measurement method of the cutterhead source position electrode array.

The measurement strategy of the electrode array determines to a certain extent the quality of the image reconstruction. Common measurement methods principally include adjacent excitation adjacent measurement, interval excitation adjacent measurement, relative excitation adjacent measurement, etc. The main difference between these measurement methods lies in the selection of the excitation electrode pair and the measurement electrode pair. Based on the adjacent-excitation–adjacent-measurement method, this study proposes a measurement method suitable for the cutterhead-source electrode array, as shown in Figure 5. Each electrode in the electrode array serves as both an excitation electrode and a measurement electrode. When beginning the measurement process, electrode No. 1 and the receiving electrode located behind the tunnel are the excitation electrodes, and the remaining electrodes are the measurement electrodes. The rules are as follows: First, the No. 1 electrode is used for excitation, and the adjacent No. 2 and No. 3 electrodes perform the potential measurement. Then, the No. 3 and No. 4 electrodes perform measurements until the No. 12 and No. 13 electrodes complete the measurement. At this point, the potential measurement work of electrode No. 1 for excitation is completed. Then, electrode No. 2 is used for excitation, and electrodes No. 3 and 4 start measuring, until electrodes No. 13 and No. 1 finish the measuring. Finally, analogously, electrode No. 13 starts excitation, electrodes No. 1 and 2 begin to measure, until electrodes No. 11 and 12 finish measuring, and the measurement of the entire electrode array is completed. It should be noted that electrode No. 4 located in the center of the electrode array does not perform a repeated role in the excitation and measurement processes.

## 3. Numerical Simulation

### 3.1. Characteristics of Focusing Electric Field

When using the 3D-ERT advanced detection method using source-position electrode excitation for the detection of undesirable geological bodies in front of the tunnel face, the excitation current will display the phenomenon of dispersion; therefore, it is impossible to accurately obtain the required geological information if the excitation current is not constrained. Aiming at the three-dimensional full-space environment during the tunnel construction process, COMSOL software (v 6.1) is used to establish a geoelectrical model for simulation, comparing and analyzing the electric field strength and current density distribution in the tunnel space when a single-point power supply and a focused power supply are used.

**Figure 6:**
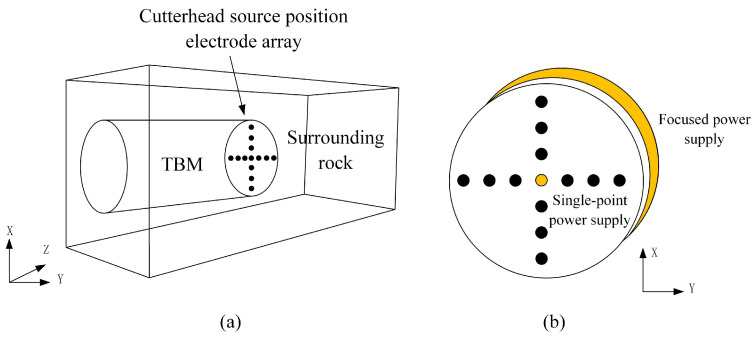
Schematic diagram of the shield tunnel advanced detection model. (**a**) Schematic diagram of the 3D model; (**b**) Cutterhead electrode array.

The established geoelectrical model is shown in Figure 6a. The dimensions are consistent with the simulation model established in Section 2.4; additionally, the electrodes are regarded as point electrodes because of their extremely small dimensions with respect to the tunnel-boring machine. As shown in Figure 6b, in the case of a single-point power supply, the electrode at the center of the electrode array is set as the supply electrode, and the supply current is 1A; in the case of a focusing power supply, in addition to the supply electrode at the center position, another circle of focusing electrodes is arranged at the front edge of the TBM shield, and the current size is also 1A. The other electrodes in the electrode array, except for the supply electrode, are used as the measurement electrodes, and the background resistivity of the surrounding rock is set to be 1000 Ω·m.

**Figure 7:**
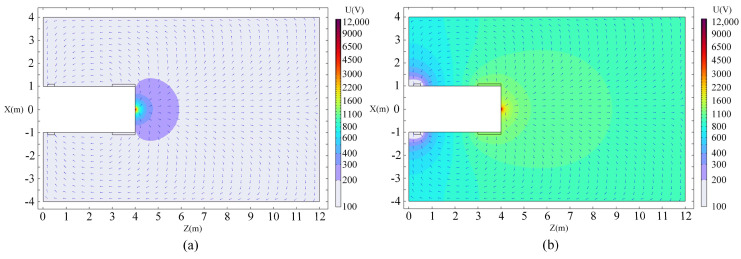
Electric field distribution map. (**a**) The single-point power supply; (**b**) The focused power supply.

The characteristics of the electric field in the detection area in front of the shield-tunnel face are analyzed, including an electric field distribution map and a potential distribution cloud map. The E-field distribution map can clearly show the relationship between the E-field strength and the depth change under two different modes of single-point power supply and focused power supply, while the potential distribution cloud map reflects the level of electric potential, and the direction and length of the arrows in the map indicate the direction and size of the current, respectively. The slicing operation is performed at Y = 0 m, and the electric field distribution map is obtained, as shown in Figure 7.

Observing the electric field distribution diagram, we can see that the current spreads around at the cutterhead power supply electrode, The potential is higher near the power supply electrode and decreases with the increase in distance. Comparing the electric field distribution characteristic diagrams of the two, it is discovered that when there is no focusing electrode and only a single point of power supply, the electric field shows a semicircular distribution; specifically, the voltage value starts from the power supply electrode and gradually decreases along the front of the tunnel face. This is due to the fact that the excitation current is only applied to the supply electrode at the center of the cutterhead and the scope of the electric field formed is relatively small. When there is a focusing electrode for focusing the power supply, according to the principle of the mutual repulsion of electrodes with the same polarity, the current is constrained and concentrated to propagate in front of the tunnel face, thus making the detection distance larger.

**Figure 8:**
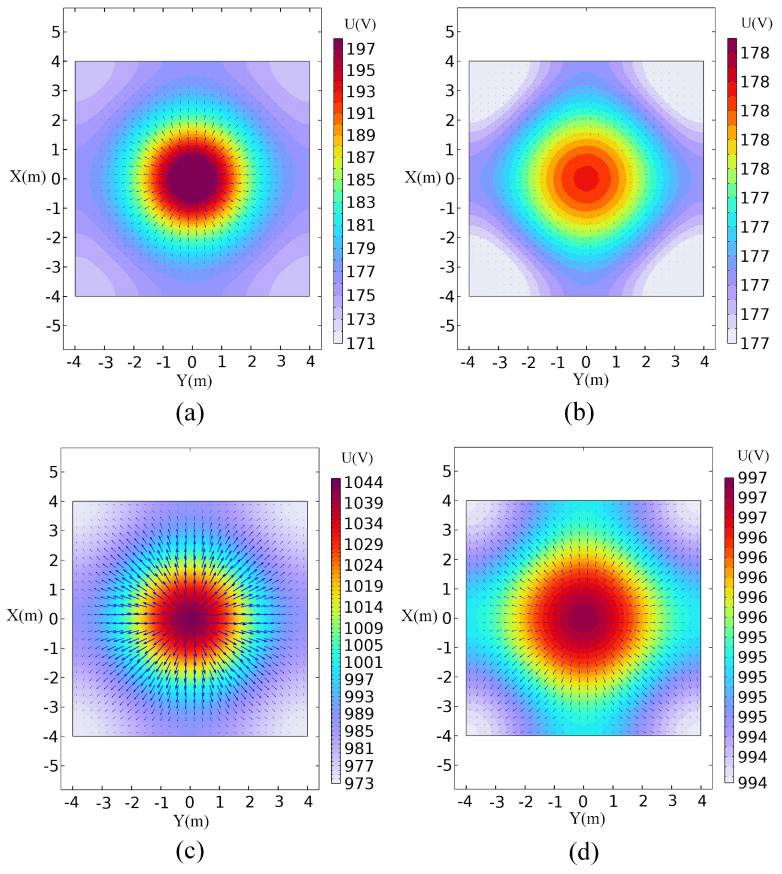
Potential slice diagram. (**a**) The single-point power supply Z = 6 m; (**b**) The single-point power supply Z = 10 m; (**c**) The focused power supply Z = 6 m; (**d**) The focused power supply Z = 10 m.

Potential cloud diagrams are extracted at depths Z = 6 m and Z = 10 m, as shown in Figure 8. Observing the potential slice diagrams of the two at different positions, it can be observed that during the advanced detection process of the shield tunnel, along the tunnel excavation direction, if the distance between the node potential and the cutterhead supply electrode increases, the node potential value decreases.

Figure 8a,c show the potential slice plots at Z = 6 m position for both single-point and focused power supply modes, with the largest potential values at the center of the slices, 197 V and 1044 V, respectively. In addition, there is a rapid decrease in the potential value along the radial direction of the tunnel for the single-point power supply mode, a higher potential inside the tunnel, and a gradual decrease in the external potential for the focused power supply mode. Figure 8b,d, respectively, show the potential slice diagrams of the two at Z = 10 m. It can be seen from the figure that the highest potential values are 178 V and 997 V, respectively. When the node potential moves from the center of the cutterhead to the radial direction of the tunnel, the potential value shows a changing trend from high to low. In Figure 8, overall, it can be seen that the current densities are larger in the vicinity of the cutterhead supply electrodes and disperse around; the current density gradually decreases with the increase in the distance of the supply electrode; and in the same slice position, the current is larger in the focused power supply than in the single-point power supply. In summary, the electric field spreads more slowly when the supply is focused, indicating that the use of focused electrodes to constrain the current is beneficial for the detection of more distant areas.

### 3.2. Inversion Imaging of Undesirable Geologic Bodies

**Figure 9:**
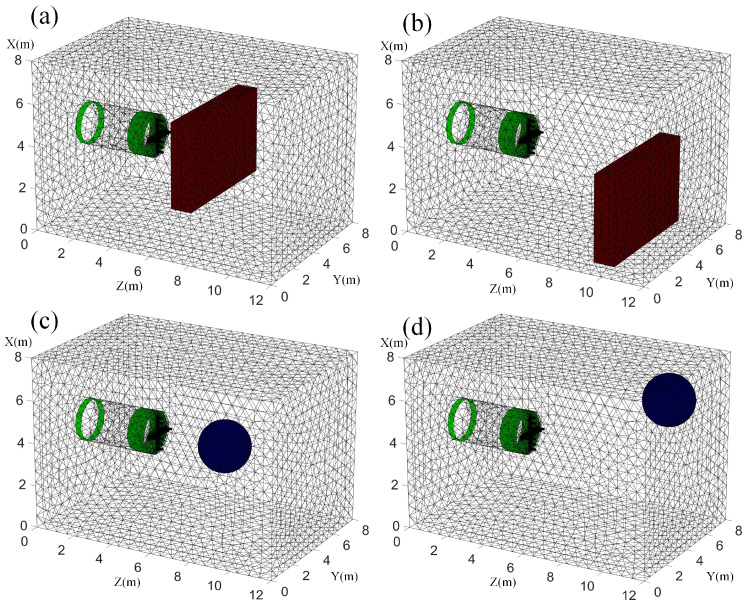
Simulation model based on the finite-element method. (**a**,**b**) are rectangular high-resistance anomalous bodies; (**c**,**d**) are spherical low-resistance anomalous bodies.

The finite-element method (FEM) is used to construct the three-dimensional forward model, and when image reconstruction is carried out, the forward model is dissected into networks. Then, the network density of the surrounding rock is selected to be more refined, and the network density of the TBM model and the undesirable geological body is selected to be ultra-refined, taking into account the requirements of calculation accuracy and saving in calculation time. The size of the forward model is consistent with the simulation model established in Section 2.4. According to the actual situation, the undesirable geological bodies existing in front of the tunnel face are simplified into two models, which are a rectangular high-resistance anomalous body simulating faults and a spherical low-resistance anomalous body simulating water-bearing caverns, as shown in Figure 9a,b representing the rectangular anomalous body and Figure 9c,d representing the spherical anomalous body. These anomalous bodies are distributed in front of the tunnel face. The dimensions of the rectangular anomalous body are 4 m × 4 m × 0.5 m, and one is located at the center axis of the tunnel at 3.5 m proximal to the tunnel face, and the other is located at the lower left of 6 m distal to the tunnel face; the radius of the spherical anomalous body is 1.2 m, located at 3.5 m proximal to the tunnel face and 6 m distal to the upper right of the tunnel face, respectively.

Figure 9 shows the simulation model based on the finite-element method. Under the simulation conditions, the conductivity of the background is set to 500 Ω·m, where the conductivity of the low-resistance anomalous body and the high-resistance anomalous body are set to 5 Ω·m and 1000 Ω·m, respectively. The magnitude of the excitation current is 1A. The GREIT algorithm is used to execute the image reconstruction, and the reconstructed portion is the unknown area in front of the tunnel face.

**Figure 10:**
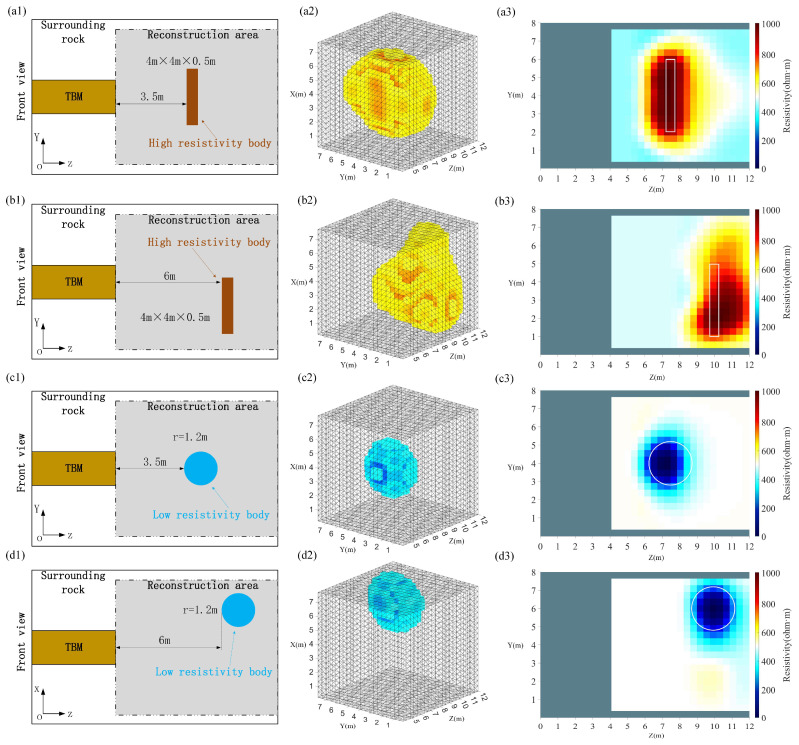
Simulation model and reconstructed results. The (**a1**–**d1**) represent a schematic of the location of the high-resistance anomalous body and the low-resistance anomalous body; the (**a2**–**d2**) represent a map of the 3D reconstruction results; the (**a3**–**d3**) represent a 2D sliced image passing through the center of the anomalous body along the *Y*-axis.

As shown in Figure 10, two models with a total of four different scenarios were created to assess the feasibility of this study, with each row corresponding to one scenario; the first column contains a schematic of the location of the high-resistance anomalous body and the low-resistance anomalous body; the second column provides a map of the 3D reconstruction results; and the third column is a 2D sliced image passing through the center of the anomalous body along the *Y*-axis. The image reconstructed through the GREIT algorithm will magnify the location of the sensitive area. Observing the three-dimensional reconstruction results, for the high-resistance anomalous body, the reconstructed shape deviation of the rectangular high-resistance anomalous body located at the lower left of the far end of the tunnel face is large; for the low-resistance anomalous body, its overall reconstruction effect is better than that of the high-resistance anomalous body, and its location and shape can be clearly observed. Observing the two-dimensional slice diagram, we can clearly see the shape and location of the anomalous body. Compared with the low-resistance anomaly, the reconstruction result relating to the sensitive area of the high-resistance anomaly is amplified to a higher extent. The possible reason is that the 3D-ERT advanced detection method is more sensitive to low-resistance anomalous bodies. From the 3D reconstruction results of the high-resistance anomalous bodies, it can be seen that their overall shapes are closer to a disk, which may be due to the fact that the voxel model reconstruction results are not prominent for the diagonal features. In general, the position and shape of the undesirable geological bodies in front of the tunnel face have been well reconstructed. The numerical simulation results further validate the feasibility of the 3D-ERT advanced detection method using the source-position electrode excitation proposed in this study, which is further verified with the laboratory physical simulation experiments in the following section.

## 4. Physics Simulation Experiment

### 4.1. Platform Building

In the electrical-resistivity method, physical experiments are usually used to simulate complex construction environments. There are three main experimental methods used in physical-simulation experiments: the flume-simulation experiment, the soil-tank-simulation experiment, and the conductive-paper-simulation experiment. The soil-tank-simulation experiment is suitable for situations where undulating terrain and anisotropic media need to be constructed, and the conductive-paper-simulation experiment is suitable for situations where only electric field distribution is discussed. In view of the fact that the laboratory environment cannot fully reproduce the physical conditions of the engineering site, it is assumed that the geological bodies with different conductivities in front of the tunnel face in actual working conditions can be divided into two categories: high resistance and low resistance, and the soil medium in front of the tunnel face is considered to be both uniform and isotropic. In this study, a flume-simulation experiment was chosen to study the detection effect of the proposed method in the case of isotropic media. In the flume-simulation experiment, the water itself representing the surrounding rock is uniform and is suitable for simulating the conditions of different anomalies in a uniform isotropic medium.

**Figure 11:**
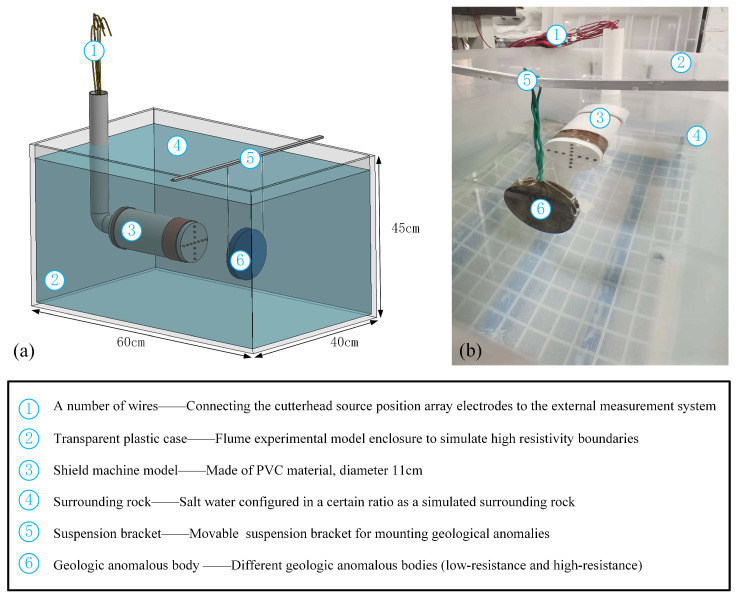
Schematic diagram of flume simulation experiment platform. (**a**) flume experiment model, (**b**) indoor experiment model.

The schematic diagram of the built flume-simulation experimental platform and the layout of the indoor experimental model are shown in Figure 11. The geometric similarity ratio of the experimental model is 1:100. The size of the water tank is 60 cm × 40 cm × 45 cm, the height of the water surface is 40 cm, and a certain proportion of salt water is configured to simulate the surrounding rock. The simulated TBM model consists of PVC hollow pipes, copper electrodes, and several wires. The PVC hollow pipe simulates the TBM fuselage during the excavation process. A hoop of ring-shaped copper sheets is attached to the front end of the PVC hollow pipe as a simulated shield, and the ring-shaped copper sheets are used as the focusing electrodes. Considering that electrical insulation is required between the simulated cutterhead and the electrode, a PVC round cover is used as the simulated cutterhead, and hot melted glue is used to fill the space between the simulated cutterhead and the PVC hollow pipe. A cross-shaped electrode array is installed on the simulated cutterhead, with an electrode diameter of 0.4 cm and an electrode spacing of 1 cm. Another ring-shaped copper sheet is arranged behind the PVC hollow pipe as a receiving electrode, and waterproof cement is used to fill the gaps around each electrode to prevent water leakage. Above the water tank, an experimental box is set up with a mounting suspension, which is used to hang the undesirable geological bodies. Wires are used to connect the electrode array and the measurement system to collect data.

### 4.2. Experimental Design

**Figure 12:**
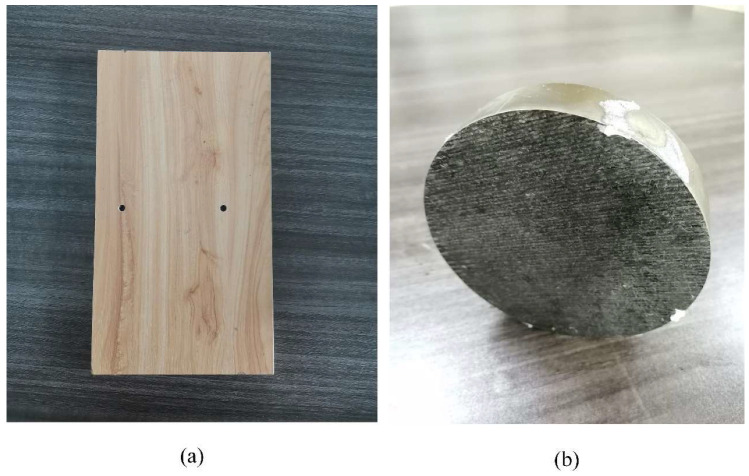
Two different anomalous bodies. (**a**) High-resistance wooden board; (**b**) Low-resistance metal discs.

In this experiment, two different foreign objects were selected as the anomalous body in front of the tunnel face. To evaluate the feasibility of this study, the two anomalous bodies were a high-resistance wooden board and low-resistance metal discs. The resistivity ratio of salt water to wooden boards and metal disks is similar to the ratio of the actual resistivity of surrounding rock to the resistivity of water-bearing caves and faults. The high-resistance wooden board had a length of 20 cm, a width of 10 cm, and a thickness of 1.5 cm, and the low-resistance metal discs had a diameter of 9 cm and a height of 2.5 cm. The two anomalous bodies are shown in Figure 12. During the experiment, the TBM model was placed in the middle of the left side of the experiment box, and the anomalous bodies were placed 15 cm from the proximal end of the TBM model and 30 cm from the distal end of the TBM model, which was on the same axis with the TBM model. The measurement strategy proposed in Section 2.5 was used for data collection.

### 4.3. Analysis of Results

**Figure 13:**
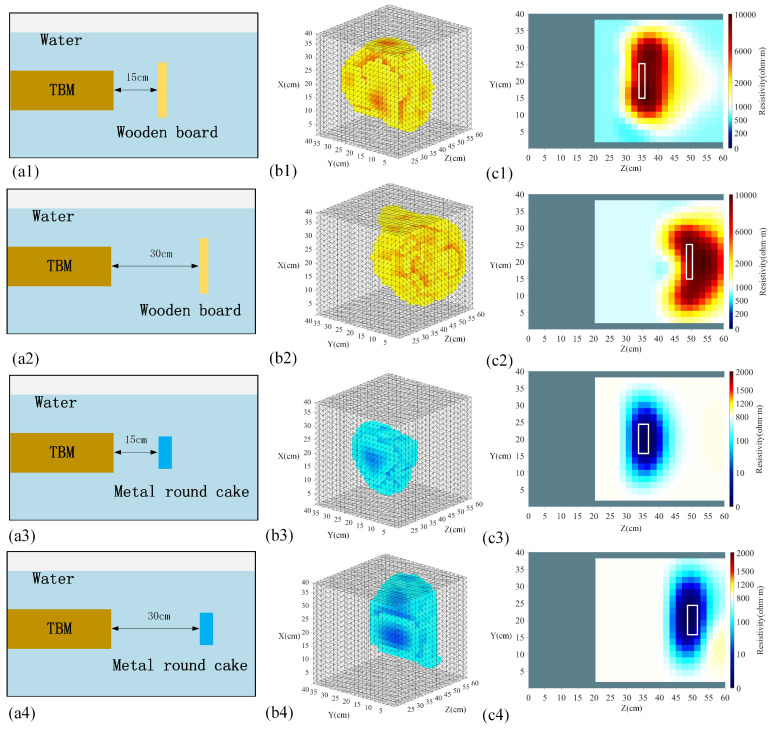
Schematic of the location of the anomalous body and image reconstruction results. The (**a1**–**a4**) represent a schematic of the anomalous body location, the (**b1**–**b4**) represent 3D reconstruction results, and the (**c1**–**c4**) represent a 2D slice map along the *Y*-axis.

In this study, a total of four scenarios of two different anomalous bodies were subjected to a flume-simulation experiment, and the complete voltage data collected were transferred to a PC for inverse imaging via the GREIT algorithm. The reconstruction results are shown in Figure 13.

In Figure 13, the first column is a schematic diagram of the location of the tested anomalous body, and the second and third columns show the 3D reconstruction results and the 2D slices along the *Y*-axis, respectively. For the high-resistance wooden board, from the analysis of the reconstruction results from two different viewing angles, when located 15 cm proximal to the tunnel face, it can be accurately reconstructed. However, the reconstructed shape of the high-resistance wooden board located 30 cm from the tunnel face changes significantly and contains more noise. This may be due to the fact that the remote abnormal body is located in a low-sensitivity region and it is difficult to obtain good inversion results. For the low-resistance metal discs, the 3D reconstruction results and the 2D slice diagrams show that the reconstruction position and shape are basically consistent, containing relatively less noise, and their reconstruction results are less amplified for the sensitive areas than the high-resistance wooden board. In general, both the foreign objects located proximally and distally to the tunnel face can be detected. The position and shape are essentially close to those of the actual ones, and the reconstruction effect of the low-resistance metal round cake is better than that of the high-resistance wooden board, which is consistent with the numerical simulation. Practice has proved that this method can effectively identify the abnormal body in front of the tunnel face.

**Figure 14:**
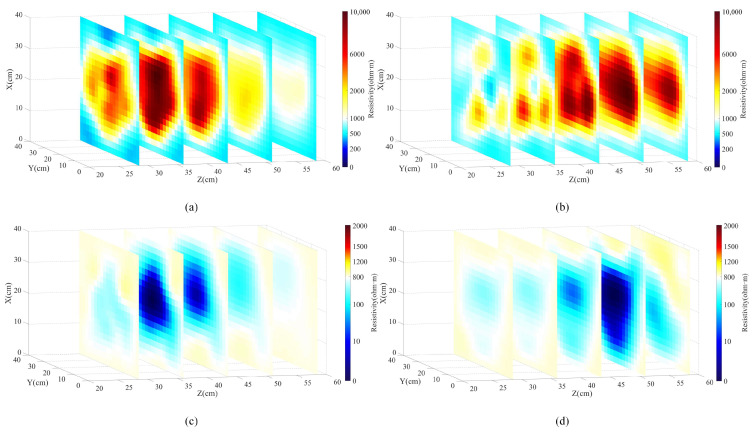
Reconstruction results after multilayer slicing along the *Z*-axis. (**a**) is the high-resistance wooden board at the proximal end of the tunnel face; (**b**) is the high-resistance wooden board at the distal end of the tunnel face; (**c**) is the low-resistance metal disc at the proximal end of the tunnel face; and (**d**) is the low-resistance metal disc at the distal end of the tunnel face.

Figure 14 is the result of multi-layer slicing along the *Z*-axis for the three-dimensional reconstructed views of the four different experimental scenarios. It can be seen from the figure that if the slice passes through the position of the abnormal body, its size and shape can be more accurately displayed. According to the location of the slices, we can make a clearer judgment of the location of the anomalous body, which will make the visualization of the undesirable geological body in front of the tunnel face more accurate. From the slice images of groups (a) and (b), we can observe that the reconstructed positioning of the high-resistivity wooden board is accurate, and the resistivity is obvious at 15 cm and 30 cm in front of the tunnel face; however, its shape is distorted and the recognition accuracy is relatively low. From the slice maps of groups (c) and (d), the low-resistance metal discs can be clearly observed, and their accuracy is close to the real situation, with the shape conformity of the imaging results near the proximal end of the tunnel face being higher than that of the distal end. From the overall multi-layer slices, drag shadows appear at the back of both the high-resistance wooden board and the low-resistance metal disc slices, and the drag shadows of the high-resistance wooden board are more evident. This is due to the relatively larger size of the high-resistance wooden board, which shows a larger-scale high-resistance response, whereas the low-resistance metal discs have relatively low resistance and size, and the current passes through them with stronger penetration, so their imaging results are better.

### 4.4. Reconstructed Image Evaluation

Image evaluation can reflect the overall reconstruction quality of the image. This article evaluates the reconstructed image through two indicators: location error (LE) and shape error (SE). First, define the set of unit pixel values greater than one-quarter of the maximum pixel value in the reconstructed image as the region of interest set, denoted as $\Omega_{P}$. The location error function LE is as follows:

(15) LE=dPre−dRel

In the formula, $d_{\mathrm{Pr}e}$ is the distance from the preset target to the center point in the reconstructed image, $d_{Re}$ is the distance from the reconstructed target to the center point in the reconstructed image, and l is the length of the reconstructed area. The smaller the LE value, the smaller the position error.

The shape error function SE is as follows:

(16) $$SE=\frac{\left|A_{\Omega \subset P}-A_{\mathrm{Pr}e}\right|}{A_{\mathrm{Pr}e}}$$

The calculation method of the shape error is the change of the unit area $A_{\Omega \subset P}$ of the region of interest in the reconstructed image relative to the projected area $A_{\mathrm{Pr}e}$ of the preset target on the XOZ section. The closer the SE value is to 0, the smaller the shape error is.

**Figure 15:**
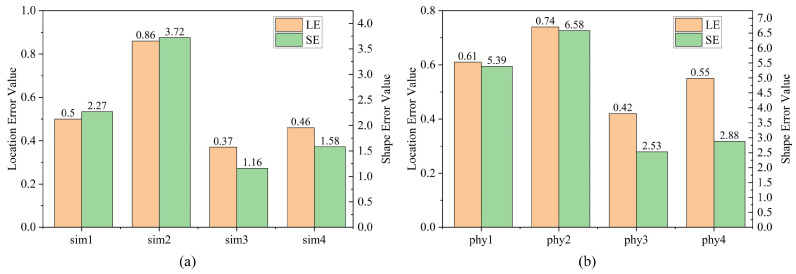
Image reconstruction quality evaluation results. (**a**) Reconstruction quality evaluation results of simulation experiments, (**b**) Reconstruction quality evaluation results of physical experiments.

The image evaluation index is used to evaluate the reconstruction results of the simulation experiment and physical experiment, and the calculation is performed using Equations (15) and (16). The reconstruction quality evaluation results of the simulation experiment are shown in Figure 15a. In the figure, sim1 represents the rectangular high-resistance abnormal body located 3.5 m near the tunnel face, and sim2 represents the rectangular high-resistance abnormal body located 6 m below the far end of the tunnel face. sim3 and sim4 represent the spherical low-resistance abnormal bodies located at the proximal and distal ends of the tunnel face, respectively. It can also be seen that the spherical low-resistance abnormal body near the tunnel face has the smallest LE and SE values, indicating that its reconstruction quality is high. The reconstruction quality of the spherical low-resistance abnormal body at the far end of the tunnel face is slightly higher than that of the rectangular high-resistance abnormal body at the proximal end of the tunnel face. The LE and SE values of the rectangular high-resistance abnormal body located at the far end of the tunnel face are relatively large, indicating that the reconstruction quality is slightly lower.

The reconstruction quality evaluation results of the physical experiment are shown in Figure 15b. Phy1 and Phy2 represent the high-resistance wooden boards located at the proximal and distal ends of the tunnel face, respectively, and Phy3 and Phy4 represent the low-resistance metal discs located at the proximal and distal ends of the tunnel face, respectively. On examining Figure 15b, the LE and SE values of the low-resistance metal discs are lower than those of the high-resistance wooden board, indicating that the reconstruction quality of the low-resistance metal discs is better than the high-resistance wooden board. The overall position error between the low-resistance metal round cake and the high-resistance wooden board is small. The shape error of the high-resistance wooden board is larger than that of the low-resistance metal disc, which means that its reconstruction quality needs to be improved.

Overall, the reconstructed image has a high position and good shape accuracy, and the reconstruction quality of the low-resistance anomaly body is better than that of the high-resistance anomaly body. This shows that the 3D-ERT advanced detection method using source-position electrode excitation for tunnel-boring machine source electrode excitation is correct and will help promote the further development and innovation of shield-tunnel-related technologies.

## 5. Conclusions

In this study, a 3D-ERT advanced detection method using source-position electrode excitation of a tunnel-boring machine is proposed, which is more suitable for the complex geological environment of tunnel construction using boring machines, and does not affect the normal construction of tunnels during the process of detection. In addition, this method can accurately describe the size, morphology, and location of the undesirable geological body in front of the tunnel face, and the feasibility and effectiveness of the method can be verified through a series of numerical simulation experiments and indoor physical simulation experiments. The following conclusions are drawn: (1) Combined with the characteristics of shield-tunnel construction, a cutterhead source electrode array was designed, and a three-dimensional geoelectrical field model was constructed to study the different arrangements of the electrode array. The results show that compared with the one-line electrode array, the cross-shaped electrode array can eliminate the symmetry effect and avoid artifacts on the other side of the imaging target. (2) A geoelectrical model was established to study the characteristics of the focused electric field. The analysis concluded that a focused supply can constrain the excitation current and facilitate detection in farther areas. At the same time, four three-dimensional finite-element models were established to conduct research into the inversion imaging of abnormal bodies. The research results show that abnormal bodies with different positions and shapes can be well reconstructed. For a high-resistance abnormal body far away from the tunnel face, the reconstruction effect is somewhat insufficient, and the reconstruction effect of a low-resistance abnormal body is better than that of high-resistance anomalies. (3) A laboratory flume-simulation experimental platform was built to study the imaging results of two different foreign objects in four scenarios. Through the reconstruction results, the location and shape of the foreign objects can be intuitively perceived. It was also found that the recognition of low-resistance foreign objects is higher than that of high-resistance foreign objects, which is consistent with the results of numerical simulation experiments. The feasibility and effectiveness of the proposed method were verified, and thus the method provides a new way of thinking about the detection of the undesirable geological body to tunneling with excavation without stopping the tunneling work. However, for different kinds and scales of geological anomalous bodies in the complex and changeable tunnel environment, it is necessary to combine with other over-advanced geological forecasting methods to carry out and ensure comprehensive detection.

## Data Availability

The original contributions presented in the study are included in the article, further inquiries can be directed to the corresponding author.
